# Inconsistency in Fluid Balance Calculations in UK Paediatric Intensive Care Units: A Short Research Report

**DOI:** 10.1111/nicc.70516

**Published:** 2026-05-14

**Authors:** Lyvonne N. Tume, Lindsay Kenworthy, Angela Aramburo

**Affiliations:** ^1^ Edge Hill University Ormskirk UK; ^2^ Alder Hey Children's NHS FT Liverpool UK; ^3^ PICU, Alder Hey Children's NHS FT Liverpool UK; ^4^ Royal Brompton Hospital, Guy's & St Thomas' NHS Foundation Trust London UK; ^5^ Imperial College London London UK

**Keywords:** child, critical care, fluid balance chart, fluid calculations, neonate

## Abstract

In critically ill children, an accurate fluid balance record is important, especially in small children. Before a multicentre trial begins, we wanted to ascertain what fluids nurses included in their intake across UK Paediatric Intensive Care Units (PICUs). A cross‐sectional electronic survey was sent to all UK Paediatric Intensive Care Units (PICUs) nurse educators. Thirty responses representing 23 PICUs revealed substantial variation, with many units excluding flushes after intravenous or enteral drugs and very few recording flushes given after blood sampling or blood gas analysis. In many PICUs, fluid balance charts predominantly captured intravenous infusions and drugs only. These omissions risk unrecognised ‘fluid creep’ and highlight a clear target for nurse education before conservative fluid management trials.

## Impact Statements

- What is known about the topic
  ○ Ensuring an accurate fluid balance record is essential in critically ill children.
- What this paper adds
  ○ This survey of UK PICUs practice shows variation in the fluids recorded in this intake.
  ○ It also suggests the move to electronic health records for fluid balance charting has changed the skills of nurses in this daily calculation.

## 1 Introduction

Intravenous (IV) fluid use in critical care is universal, yet there is no clear consensus on optimal fluid management strategies in critically ill children. Initial resuscitation is often lifesaving, but children subsequently receive substantial volumes of other fluids via IV drugs, maintenance solutions, enteral medications and line flushes, all of which can contribute to clinically important ‘fluid creep’, particularly in small infants [1, 2, 3]. PICUs typically admit children aged 0–17 years, with the majority under 2 years of age [4]. Unlike adults, children (especially those under 10 kg) have a much higher total body water (80%) compared to adults at 60%, more of which is extracellular, and fluids are calculated per body weight (kg) [5]. Small children, especially neonates, are at far greater risk of fluid overload, thus a highly accurate fluid intake and output record (called a fluid balance chart) is essential [5].

Fluid accumulation is associated with worse outcomes, including prolonged mechanical ventilation, longer intensive care unit stay and higher mortality in critically ill children [6, 7, 8]. To address this uncertainty, the multicentre PIVOTAL platform trial [9], which includes a conservative fluid management domain, is scheduled to commence in the United Kingdom in 2026. Ahead of trial recruitment, we sought to understand current practice in PICU fluid balance charting (FBC), specifically which fluids are included by nurses and how this might highlight areas for targeted nurse education.

## 2 Aim

To describe which fluid inputs UK PICU nurses include in their fluid balance calculations, and to explore how nurses calculate daily fluid intake.

## 3 Methods

A cross‐sectional electronic survey using Microsoft Forms was distributed to all UK PICU nurse educators via the UK Paediatric Critical Care Society‐Educators Group (PCCS‐E) network in December 2025, with one response requested per PICU. Nurse educators were targeted because they train bedside nurses and develop local procedural guidance. The survey was designed by two content and clinical experts (LT, AA) and pilot tested for face validity with one UK PICU nurse educator (LK); no amendments were required. The final survey contained four questions (three multiple‐choice and one free‐text open question) and was disseminated via email and the PCCS‐E NHS Futures site. The study is reported in line with the CHERRIES checklist [10]. The survey was analysed by simple descriptive statistics and free‐text response by simple thematic analysis [11].

## 4 Results

Thirty responses were received from 23 of 26 UK PICUs. Duplicate responses were reviewed for inconsistencies; where conflicting data were identified, clarification was sought and the inaccurate response removed, leaving data from 23 PICUs for analysis. This represents 95% of PICUs intending to participate in the forthcoming NIHR HTA‐funded PIVOTAL trial [9].

**FIGURE 1:**
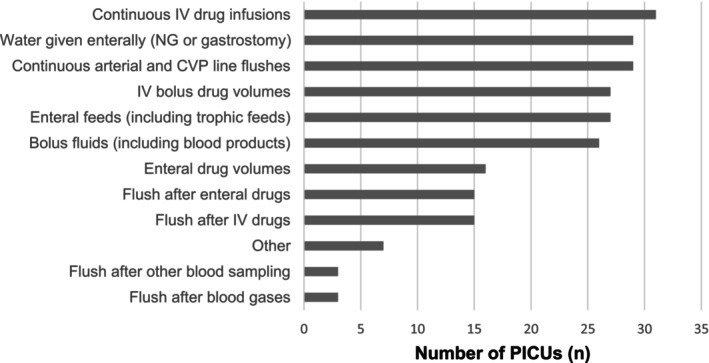
Fluids included in PICU fluid intake calculations across 23 UK units (multiple responses per unit allowed). CVP, central venous pressure; IV, intravenous; NG, nasogastric.

**FIGURE 2:**
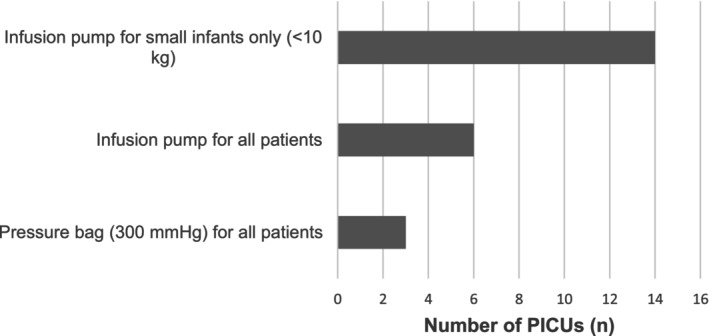
Methods used to deliver continuous arterial and central venous pressure line flushes across 23 UK paediatric intensive care units.

All units reported including intravenous drug infusions, intravenous drugs, pressure line flushes, enteral feeds and enteral water in their fluid balance charts (Figure 1). Fewer units included saline flushes after intravenous bolus drugs or enteral medications, and flushes given after blood sampling and blood gas analysis were rarely recorded. Fourteen of 23 (61%) PICUs used a weight threshold (typically < 10 kg) below which arterial and venous continuous flush solutions were delivered via infusion pumps with recorded volumes, whereas three of 23 (13%) used pressure bags inflated to 300 mmHg with volume estimated rather than measured (Figure 2).

Free‐text responses describing how nurses calculated daily fluid intake highlighted some common practices but also notable variation. All units reported that fluid balance was calculated each shift or day against the prescribed total fluid allowance. However, some units described subtracting only intravenous infusions and intravenous drugs from this allowance to determine the remaining volume available for enteral nutrition or maintenance fluids, whereas others also included drug flushes; one unit reported not counting trophic feeds as part of fluid intake.

Respondents also described patient‐specific adaptations to practice, for example:

For post‐op cardiacs the input is very strict (drug flushes all included) but for others with issues around nutrition or poor growth this can be overridden by the medics and dieticians.

Two units expressed concerns about the accuracy of fluid balance recording following transition from paper to electronic health records. One nurse educator commented:

Initially fluids were calculated daily by nurses looking at all fluids going in and calculating the space left, but since moving to [new electronic health record] I am unsure of the accuracy of the FBC.

Another noted that ‘the use of computerised systems has brought many challenges, the skills of ICU nurses re fluid balance is being lost somewhat’.

## 5 Discussion

This national survey of UK PICU nurse educators demonstrates substantial variation in which fluid inputs are included in fluid balance calculations, despite the central role of fluid balance in the care of critically ill children. Although all units recorded major fluid sources such as intravenous infusions and enteral feeds, many did not routinely include smaller but cumulatively important volumes such as saline flushes after intravenous or enteral drugs and flushes associated with blood sampling. These omissions may result in unrecognised ‘fluid creep’, particularly in small infants, where even modest excess volumes can contribute to fluid accumulation.

The finding that many units estimate rather than measure continuous flush volumes further illustrates the challenge of achieving accurate fluid balance records. For a conservative fluid management strategy, such as that planned within the PIVOTAL trial, systematic underestimation of true intake could lead to protocol deviations, misinterpretation of trial results and, more importantly, suboptimal clinical decision‐making. Standardising what constitutes ‘recordable’ fluid intake is therefore essential. No studies were found relating to fluid balance charting in paediatric critical care. However, a systematic review of fluid balance charting in 23 studies in adult critical care [12] found that the quality of fluid balance charting was poor and inadequate.

From a nursing perspective, fluid balance charting is a core task that requires clear guidance, appropriate tools and ongoing education. Our study suggests that current guidance may be incomplete or interpreted variably, particularly regarding small‐volume fluids and flushes. It appears that the change to electronic health records has changed the way fluid balance intakes and outputs have been recorded, with a perception of potential inaccuracy and loss of nursing skill in this calculation. Nurse educators are well placed to lead harmonisation of practice by updating local policies, embedding standard definitions of fluid inputs within education programmes and ensuring that electronic or paper charts facilitate comprehensive recording.

The study has several limitations. It relies on self‐report from nurse educators and may not fully capture bedside practice, and the survey included a limited number of questions, which restricted exploration of contextual factors. Nonetheless, the high response rate from PICUs intending to participate in the PIVOTAL trial provides a useful overview of current UK practice.

## 6 Conclusion

There is marked inconsistency in which fluids are included in fluid balance intake calculations across UK PICUs, with omission of flushes after drugs, blood sampling and other small‐volume inputs potentially leading to substantial hidden fluid intake and fluid creep, especially in neonates and infants. The move to electronic health records has changed the way fluid balances are recorded. While some aspects of accuracy may have improved, systems still require manual nursing adjustments of infusion rates or manual addition of flush and drug volumes, thus remaining prone to error, with some nurses perceiving a loss of calculation skills associated with reduced manual charting. These findings support the need for standardised guidance, targeted nurse education and careful configuration of electronic charting to ensure accurate, nurse‐led fluid balance management in future clinical practice and research.

## Funding

The authors have nothing to report.

## Disclosure

We declare no AI has been used in this study or in the writing of this manuscript.

## Ethics Statement

Approval from the society was gained.

## Conflicts of Interest

Two of the authors (L.N.T. and A.A.) lead the Fluid domain of the NIHR HTA‐Funded Platform trial, but no funding was used for this work. L.K. has no COI to declare.

## Data Availability

The data that support the findings of this study are available on request from the corresponding author. The data are not publicly available due to privacy or ethical restrictions.
